# Entomophagy Attitudes Among Turkish Generation Z University Students: A Scale Validation and Path Analysis Model for Sustainable and Healthy Dietary Choices

**DOI:** 10.1002/fsn3.70397

**Published:** 2025-06-08

**Authors:** Emre Duman, Alev Keser

**Affiliations:** ^1^ Faculty of Health Sciences, Department of Nutrition and Dietetics Siirt University Siirt Türkiye; ^2^ Faculty of Health Sciences, Department of Nutrition and Dietetics Ankara University Ankara Türkiye

**Keywords:** edible insects, entomophagy, food choice behavior, generation Z attitudes, healthy diet, sustainable eating

## Abstract

The global challenges of warming temperatures, reduced rainfall, and extreme weather events have heightened the need for sustainable food sources. With a growing world population, there is an increasing demand for environmentally sustainable food production methods, making edible insects a promising alternative due to their nutritional and ecological benefits. This study was conducted to validate the Turkish version of the Entomophagy Attitude Questionnaire (EAQ) and examine, through path analysis, the relationship between university students' attitudes toward insect consumption and sustainable, healthy eating behaviors. The sample consisted of 641 Generation Z students aged 18–23. Data were collected via the Sustainable and Healthy Eating Behaviors Scale (SHEBS), the EAQ, and a demographic survey conducted through Google Forms. Results indicated that the EAQ is a reliable and valid tool, with significant correlations among its components (*p* < 0.05). Among SHEBS dimensions, the “healthy and balanced diet” factor scored the highest (mean: 4.744), while the “local food” factor scored the lowest (mean: 3.361). Path analysis showed an acceptable model fit for the SHEBS dimensions on the EAQ, with “seasonal foods and avoiding food waste” showing a positive effect on the “interest” dimension of entomophagy (*p* < 0.05); however, no significant effect was observed for the “disgust” dimension (*p* > 0.05). The study suggests that public awareness should be raised to reduce negative attitudes toward entomophagy. Future studies should develop comprehensive interventions to overcome cultural barriers to entomophagy and promote this alternative to a wider audience.

## Graphical abstract

This study validates a Turkish adaptation of the Entomophagy Attitude Questionnaire and employs path analysis to explore how sustainable dietary practices influence entomophagy attitudes among Turkish Generation Z university students. The findings highlight a significant positive association between sustainable eating behaviors and interest in edible insects, offering insights for promoting alternative, healthy protein sources in Türkiye.

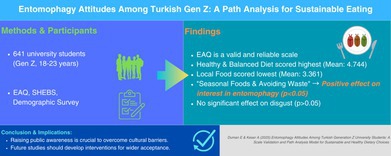

## 1 Introduction

Global warming and climate change are among the most important environmental problems of our time and constitute two of the greatest threats facing our planet (IPCC 2023). While the rise in the earth's temperature due to increasing greenhouse gas concentrations is defined as “global warming” (Al‐Ghussain 2019), the long‐term variation in weather conditions is called “climate change” (Bhattacharya 2019). Human‐induced activities such as the use of fossil fuels are considered among the main causes of climate change, which negatively affects the ecological balance of our planet (Rahmani and Ahmadi 2024). For example, between 2011 and 2020, the global surface temperature increased by 1.1°C compared to the period 1850–1900 (IPCC 2023).

Total global greenhouse gas emissions are greatly influenced by human activities such as the burning of fossil fuels, deforestation, and industrial processes. In 2023, global CO_2_ emissions reached 35.8 Gt, showing a slight increase of 0.1% compared to 2022 (Liu et al. 2024). This trend is expected to continue in the coming years (Friedlingstein et al. 2024). It is observed that human activities continue to be the main source of these emissions and contribute to climate change by changing the Earth's energy balance (Rahmani and Ahmadi 2024).

As the world population continues to grow, the environmental impacts of this increase are becoming more evident. According to the UN Department of Economic and Social Affairs, the world population is expected to reach 8.5 billion in 2030 and 9.7 billion in 2050 (United Nations, Department of Economic and Social Affairs, Population Division 2022). This situation creates serious pressure on existing resources and further increases the need for sustainable food production. Although enough food is produced in the world to feed everyone, millions of people still struggle with chronic hunger (Fanzo et al. 2021). At the same time, health problems such as obesity are increasingly common, and this situation stands out as an indicator of inequality in food distribution (Toorang et al. 2024). In this context, the search for sustainable nutrition sources plays a critical role in the fight against global warming and climate change (Macdiarmid and Whybrow 2019).

Entomophagy (insect consumption), an innovative solution that can contribute to sustainable nutrition models, attracts attention with both environmental and nutritional benefits. Entomophagy has been used as a traditional practice in various parts of the world as a food source by humans for thousands of years (Liceaga 2022). Due to their high nutritional value and low environmental impact, insects can make important contributions to sustainable diets (OrdoñezAraque and EgasMontenegro 2021). However, although general attitudes toward insect consumption tend to be negative in most societies, they may differ depending on demographic factors and cultural experiences. For example, a study shows that insect consumption is generally rejected as disgusting among students aged 18–24 (Orkusz et al. 2020). A study by Kamenidou et al. (2023) revealed that Generation Z university students generally have a negative attitude toward insect‐based food consumption. Tuccillo et al. (2020) observed that the reasons for the inclusion of insects in the diet were sustainability, nutritional value, and curiosity, while the reasons for the exclusion of insects from the diet were disgust, price, and uncertainty. However, Platta et al. (2024) concluded in a study that health and environmental concerns among Generation Z living in Poland created a positive trend toward the consumption of foods containing edible insects. In particular, more positive attitudes toward health and environmental issues were found to increase willingness to consume such foods (Platta et al. 2024). In Türkiye, insect consumption is rarely investigated as it is contrary to traditional dietary habits (Omuse et al. 2024). Moreover, entomophagy is further hindered by a combination of psychological, cultural, and structural barriers. First, food neophobia plays a central role; many Turkish consumers exhibit a strong aversion to unfamiliar foods, especially those perceived as unnatural or visually unappealing, which reduces their openness to insect‐based products (Bakkaloğlu 2022; Hajhamidiasl et al. 2025). Second, social conformity and descriptive norms exert significant influence in this collectivist society; individuals often perceive insect consumption as socially deviant due to the lack of peer behavioral models (Hajhamidiasl et al. 2025). Third, religious beliefs—although not necessarily based on explicit prohibitions—often act as a psychological barrier. Güneş and Özkan (2018) report that consumers with Islamic beliefs tend to reject edible insects due to perceived incompatibility with religious norms, even in the absence of clear doctrinal guidance. Finally, limited market access and lack of exposure exacerbate unfamiliarity: the few insect producers in Türkiye operate exclusively within the animal feed sector, and edible insect products are virtually absent from mainstream retail channels (Bakkaloğlu 2022). This cumulative invisibility limits both practical access and experiential familiarity, which are known drivers of food acceptability.

When the nutritional values of edible insects are analyzed, it is seen that these insects are very rich in protein, fat, and micronutrients (Rumpold and Schlüter 2013). For example, the protein quality of insects such as *Gryllus assimilis* is high, and their digestibility is comparable to other food sources (Oibiokpa et al. 2018). In addition, the fat content of insects is also noteworthy, and it is known that fat content is higher in the larval forms of some insects (Hlongwane et al. 2020). However, the nutritional value of insects can vary depending on factors such as developmental stage, sex, and diet (van Broekhoven et al. 2015; Kulma et al. 2020).

Insects are not only a source of food but also have environmental and economic benefits. A study by Oonincx et al. (2020) showed that insects produce much less ammonia compared to pigs and cattle. It was also found that extracts from insects have antioxidant and antimicrobial effects (Navarro del Hierro et al. 2020; Malm and Liceaga 2021). In addition, in a study examining the effect of cricket powder from *Gryllodes sigillatus* on the gut microbiota of healthy adults, an increase in the number of probiotic bacteria *Bifidobacterium animalis* was detected (Stull et al. 2018). However, insect consumption may also have some side effects. For example, a study conducted in Laos revealed that some individuals developed allergic reactions after insect consumption (Barennes et al. 2015). Recent studies have highlighted several health concerns associated with the consumption of edible insects. A major concern is allergenicity; proteins such as tropomyosin and arginine kinase found in insects share structural similarities with allergens found in crustaceans and mites, leading to potential cross‐reactivity in sensitized individuals. There is also evidence suggesting that primary sensitization to insect proteins can occur, even in individuals without a history of allergy. Another concern is the bioaccumulation of heavy metals such as cadmium and lead in edible insects, which is influenced by the substrates on which they are reared. For example, studies have reported varying levels of these metals in commercially available insect‐based products, highlighting the need for strict monitoring. Microbial contamination is also a potential risk, particularly if insects are not processed under appropriate hygienic conditions. Pathogens such as *Salmonella* spp. and *Listeria monocytogenes* have been detected in insect products, highlighting the importance of implementing Good Manufacturing Practices (GMP) and Hazard Analysis Critical Control Point (HACCP) systems in insect rearing and processing. These concerns may be particularly salient in the Turkish context, where consumer sensitivity to food safety is high and perceived health risks—regardless of statistical probability—may strongly influence the rejection of novel foods (Raheem et al. 2019; Hajhamidiasl et al. 2025). Addressing these risks through appropriate regulation, monitoring, and consumer education is essential to ensure the safe integration of edible insects into the human diet.

Insects stand out among alternative protein sources by offering high nutritional value, low environmental impact, and favorable production costs. Entomophagy offers an important solution strategy in the transition to a sustainable nutrition system (Teixeira et al. 2023). However, in order for this food source to be accepted, public perception needs to be changed and psychological barriers such as disgust need to be overcome (Iseppi et al. 2021). In this context, information campaigns and various social initiatives are of significant importance to increase the acceptability of entomophagy. This study aims to examine the interaction between Turkish Generation Z university students' attitudes toward entomophagy and their sustainable and healthy eating behaviors. In addition, the validity and reliability of the Turkish adaptation of the Entomophagy Attitude Questionnaire (EAQ) will be tested to provide data for future social intervention and education programs. There are a limited number of studies on attitudes toward entomophagy in Türkiye, and this study aims to fill this gap and provide new data on the acceptability of entomophagy in Turkish Generation Z. Beyond the linguistic validation of the EAQ, this study makes a novel contribution to the broader literature by empirically examining how specific sustainable eating behaviors—measured by the Sustainable and Healthy Eating Behaviors Scale (SHEBS)—predict attitudes toward insect consumption among a large sample of Turkish Generation Z university students. To our knowledge, this is the first study to apply path analysis to explore the behavioral correlates of entomophagy attitudes in this population. The structural model not only identifies enabling factors (e.g., preference for seasonal foods and reducing food waste) but also highlights nonsignificant predictors (e.g., local food consumption), thereby isolating context‐specific leverage points for future public health and sustainability interventions.

In addition, by focusing on a culturally diverse and understudied population, our study addresses a geographic and generational gap in the existing entomophagy literature, which has predominantly focused on Western contexts. This diversification offers broader insights into how cultural and behavioral patterns shape receptivity to alternative protein sources. Ultimately, our findings contribute to the development of more targeted outreach and education strategies to overcome psychological barriers—such as disgust—and promote sustainable dietary transitions.

## 2 Materials and Methods

### 2.1 Study Design

This is an analytical study aiming to examine the relationship between Turkish Generation Z university students' attitudes toward entomophagy and their sustainable and healthy eating behaviors within the framework of a cross‐sectional research design.

### 2.2 Participants

The data of this study were collected in August–November 2023, and the study was conducted with Generation Z university students (18–23 years old) studying at (blinded for review) University, Türkiye. The research was initiated following the permissions granted by these units. The study adhered to the ethical guidelines set forth by the Declaration of Helsinki. The ethical approval required for the research was obtained from the (blinded for review) University Ethics Committee with the permission dated (blinded for review).

The exclusion criteria for this study were as follows: (1) students studying outside (blinded for review) University, (2) individuals under 18 or over 23 years of age, (3) vegan or vegetarian participants, and (4) students enrolled in nutrition and dietetics, food engineering, or gastronomy programs. These nutrition‐related fields were excluded to minimize knowledge bias, as students in these disciplines are more likely to have had prior academic exposure to topics such as sustainable diets, alternative proteins, and entomophagy. Their educational background may lead to systematically different attitudes—in particular, higher awareness, lower disgust, or higher interest—compared to students from other disciplines. The inclusion of these students in the study could have biased the results and reduced the generalisability of the findings to the wider Gen Z university student population. Pilot interviews revealed that these students had a significantly higher baseline familiarity with edible insects and sustainability‐related dietary issues, which could artificially inflate interest scores and compromise the measurement of attitudes among nonexpert Generation Z university students.

Cohen's sample size calculation method was used to calculate the sample size. According to this method, the minimum sample size calculated with 95% confidence interval limits (*α* = 0.05, table value 1.96), *d* = 0.05 sampling error, *p* = 0.50, and *q* = 0.5 assumptions was determined as 383 (Cohen 2013). The study was completed with a total of 641 students by reaching a higher number of participants than the calculated minimum sample size. Although the minimum sample size calculated using Cohen's method was 383, we deliberately recruited a larger sample (*n* = 641) to increase statistical power, reduce sampling error, allow subgroup comparisons (e.g., by gender), and meet statistical requirements for reliability and exploratory factor analysis.

### 2.3 Data Collection Tools

The EAQ, the SHEBS, and an online questionnaire (Google Forms) developed by the researchers in which the socio‐demographic characteristics and eating habits of the participants were questioned were applied. As a result of the decision made by (blinded for review) University to conduct the courses by distance education (online) method due to the ongoing COVID‐19 pandemic in the 2022–2023 spring semester, the study questionnaire was applied using the online Google Forms platform. The online questionnaire includes an informed consent form on the first page, in which ethical elements such as the purpose of the research, confidentiality principles, voluntary participation, and the right to leave at any time are explained. Participants started and completed the questionnaire by ticking the “I accept” box.

#### 2.3.1 Entomophagy Attitude Questionnaire (EAQ)

The EAQ is a Likert‐type scale consisting of ten questions ranging from 1 (strongly disagree) to 7 (strongly agree) developed by La Barbera, Verneau, et al. (2021). This scale has three subscales: the Disgust subscale (EAQ‐D), the Interest subscale (EAQ‐I), and the Feeding Animals subscale (EAQ‐F). The EAQ‐D consists of five questions, with higher scores indicating a greater aversion to eating insects, with a Cronbach's alpha of 0.91. The EAQ‐I consists of three questions, and higher scores indicate greater interest in eating insects; Cronbach's alpha is 0.84. The EAQ‐F consists of two questions, and higher values from this scale are indicative of a better attitude toward feeding insects to animals, with a Spearman–Brown *p* value of 0.78 (La Barbera, Verneau, et al. 2021).

##### 2.3.1.1 Translation of EAQ Into Turkish

The original EAQ questions were independently translated into the target language, Turkish, by two faculty members with proficiency in English. These two separate translations were then handled together, and the agreement and disagreement between them were analyzed in detail. The scale translated into Turkish was discussed, and necessary corrections were made in terms of meaning, grammar, and terms used, and a Turkish scale was obtained. The scale translated into Turkish was evaluated by two different faculty members from the field of Nutrition and Dietetics in terms of field, language, and suitability, and necessary corrections were made. Then, two independent linguists translated the scale from Turkish to English to prevent possible language errors. The back‐translated scale was reexamined by the faculty members to check for errors and compare it with the original scale (Beaton et al. 2000). Although no formal pilot test was conducted, the Turkish version of the EAQ was subjected to expert review and student feedback during the translation process. The reliability of the scale was then fully assessed during the main study.

#### 2.3.2 Sustainable and Healthy Eating Behaviors Scale (SHEBS)

The SHEBS was developed by ŻakowskaBiemans et al. (2019), and the Turkish validity‐reliability study was conducted by Köksal et al. (2022). The Turkish version of the scale was found to be valid and reliable with seven factors and thirty‐two items, and the Cronbach's alpha coefficient of the scale was found to be 0.912 (Köksal et al. 2022).

The scale is a 7‐point Likert‐type scale consisting of 32 questions. In addition, the scale has seven subdimensions: quality labels (regional and organic), seasonal foods and avoiding food waste, animal welfare, meat reduction, healthy and balanced diet, local food, and low fat. In the scale scoring, each option increases by 1 point from “Never” (1 point) to “Always” (7 points).

### 2.4 Data Analyses

As the survey was administered via Google Forms, completion of all items was mandatory before submission. Consequently, the dataset contained no missing values, and no imputation or listwise deletion techniques were required.

In the statistical analysis phase of the study, firstly, “frequency analysis” was performed regarding the descriptive characteristics of the participants. Then, the scale scores of the EAQ and SHEBS used in the study were obtained. The statistics of the obtained scale scores were tabulated using mean (*x̄*), standard deviation (SD), minimum (min), and maximum (max), and the internal consistency of the scales was evaluated by Cronbach's alpha coefficient. A Cronbach's alpha coefficient of ≥ 0.60 was considered acceptable (van Griethuijsen et al. 2015). Individuals' responses to the scale items were shown with a Likert chart. For construct validity, confirmatory factor analysis (CFA) was applied with the diagonal weighted least squares (DWLS) estimation technique. The basic assumptions of the Exploratory Factor Analysis (EFA) process were evaluated with Kaiser‐Meyer‐Olkin (KMO) and Bartlett's test of sphericity. A KMO value of ≥ 0.60 is considered acceptable, and a significance level of < 0.05 is recommended for Bartlett's test of sphericity (Streiner et al. 2024). GFI, AGFI, CFI, TLI, IFI, and RMSEA were analyzed as goodness of fit indices. The criteria for a perfect fit include *χ* ^2^/df values ranging from 0 to 3; GFI, AGFI, CFI, IFI, and TLI values of ≥ 0.95; and RMSEA values of ≤ 0.05. Acceptable fit criteria are defined by *χ* ^2^/df values between 3 and 5; GFI, AGFI, and CFI values of ≥ 0.90; IFI and TLI values between 0.90 and 0.94; and RMSEA values of ≤ 0.08 (Polat et al. 2023).

Path analysis was applied to examine the effect of SHEBS on EAQ. This analysis aimed to explore how sustainable eating practices influence attitudes toward entomophagy. The maximum likelihood method was used in the path analysis. In addition, Pearson correlation analysis was used to determine the severity and direction of the relationship between the dimensions. Statistical analyses were analyzed at a *p* < 0.05 significance level. IBM SPSS Statistics 27.0 and the R program were used for the analyses. In addition, IBM SPSS Amos 25.0 software, specially designed for path analysis, was also used.

## 3 Results

Table 1 presents the demographic characteristics of the individuals (*n* = 641) who participated in the study. 25.7% of the participants were male and 74.3% were female. The mean age was 20.13 ± 1.547 years, and the age range varied between 18 and 23 years. When the participants' grade‐level distribution was examined, 34.9% were first‐year students and 34.8% were second‐year students. When the participants' living arrangements were examined, 37.4% were at home with their family, while 50.9% were in a dormitory/guesthouse (private/public). 7% of the participants had a chronic disease diagnosed by a doctor.

**TABLE 1:** General characteristics of the individuals included in the study (*n* = 641).

Variable	*n*	%
Gender		
Male	165	25.7
Female	476	74.3
What grade are you in?		
First	224	34.9
Second	223	34.8
Third	61	9.5
Four and above	133	20.8
Where you live		
At home with their family	240	37.4
At home with friends	40	6.2
Alone at home	35	5.5
In dormitory/guesthouse (Private/Public)	326	50.9
Do you have a chronic disease diagnosed by a doctor?		
Yes	45	7.0
No	596	93.0
Age (years)		
*x̄* ± SD	20.13 ± 1.547	
Median	20.00	
Min–Max	18.0–23.0	

Abbreviations: Max, maximum; Min, minimum; *n*, Number of observations; SD, standard deviation; x̄, mean.

Table 2 shows the descriptive statistics and reliability analysis of the three subdimensions of the EAQ. In the “Disgust” (EAQ‐D) dimension, the mean score was 5.903 (SD = 1.748), and in the “Interest” (EAQ‐I) dimension, a relatively low level of interest was observed with a mean score of 2.607 (SD = 2.056). In the “Feeding Animals” (EAQ‐F) dimension, the mean score was 3.502 (SD = 1.968). Cronbach's alpha coefficients were 0.947 for the EAQ‐D, 0.904 for the EAQ‐I, and 0.780 for the EAQ‐F. These analyses showed that the EAQ‐D and EAQ‐I dimensions have very high reliability, while the EAQ‐F dimension has reached an acceptable reliability level.

**TABLE 2:** Statistics of EAQ and SHEBS.

	*x̄*	SD	Min	Max	Cronbach's alpha
EAQ					
Disgust (EAQ‐D)	5.903	1.748	1	7	0.947
Interest (EAQ‐I)	2.607	2.056	1	7	0.904
Feeding Animals (EAQ‐F)	3.502	1.968	1	7	0.780
SHEBS					
Quality labels (regional and organic)	3.767	1.131	1	7	0.882
Seasonal food and avoiding food waste	4.315	1.165	1	7	0.804
Animal welfare	3.802	1.485	1	7	0.811
Meat reduction	3.223	1.400	1	7	0.719
Healthy and balanced diet	4.744	1.420	1	7	0.934
Local food	3.361	1.438	1	7	0.734
Low fat	4.619	1.504	1	7	0.806
Total Score	4.004	1.062	1	7	0.951

Abbreviations: Max, maximum; Min, minimum; SD, standard deviation; x̄, mean.

In addition, Table 2 summarizes the descriptive statistics and reliability analysis of the SHEBS score. The highest mean score on the SHEBS was recorded in the healthy and balanced diet dimension with 4.744, and the lowest mean score was recorded in the local food dimension with 3.361. The overall mean of the scale was 4.004 (SD = 1.062) with a Cronbach's alpha coefficient of 0.951; the reliability coefficients of the subscales ranged from 0.719 to 0.934. These results suggest that this scale is a reliable and valid instrument for assessing participants' general dispositions toward sustainable and healthy eating behaviors.

The basic assumptions of the EFA process of the EAQ and SHEBS scales were evaluated with KMO and Bartlett's test of sphericity given in Table 3. The results of Bartlett's test of sphericity were *χ* ^2^ = 5341.813, *p* < 0.001 for EAQ and *χ* ^2^ = 14846.883, *p* < 0.001 for SHEBS. KMO values were 0.859 and 0.94, respectively. Table 3 also presents the fit indices for the CFA results of the EAQ and SHEBS. The fit index values for the EAQ were *χ* ^2^ = 103.232, df = 32, *χ* ^2^/df = 3.226, GFI = 0.946, AGFI = 0.908, CFI = 0.975, TLI = 0.964, IFI = 0.975, and RMSEA = 0.070. For SHEBS, the fit index values were *χ* ^2^ = 1733.82, df = 444, *χ* ^2^/df = 3.905, GFI = 0.942, AGFI = 0.931, CFI = 0.911, TLI = 0.966, IFI = 0.902, and RMSEA = 0.050.

**TABLE 3:** EFA results and fit indices for CFA of the scales.

EFA			
Scale	KMO	Bartlett's test of sphericity	*p*
EAQ	0.859	5341.813	**< 0.001**
SHEBS	0.94	14846.883	**< 0.001**

CFA fit indices								
Scale	*χ* ^2^	df	GFI	AGFI	CFI	TLI	IFI	RMSEA
EAQ	103.232	32	0.946	0.908	0.975	0.964	0.975	0.070
SHEBS	1733.82	444	0.942	0.931	0.911	0.966	0.902	0.050

Abbreviations: *χ* ^2^, chi‐square; AGFI, adjusted goodness of fit index; CFA, confirmatory factor analysis; CFI, comparative fit index; df, degrees of freedom; EFA, exploratory factor analysis; GFI, goodness of fit index; IFI, incremental fit index; KMO, Kaiser Meier Olkin; RMSEA, root mean square error of approximation; TLI, Tucker–Lewis index. Bold values indicate statistically significant results at *p* < 0.05.

**FIGURE 1:**
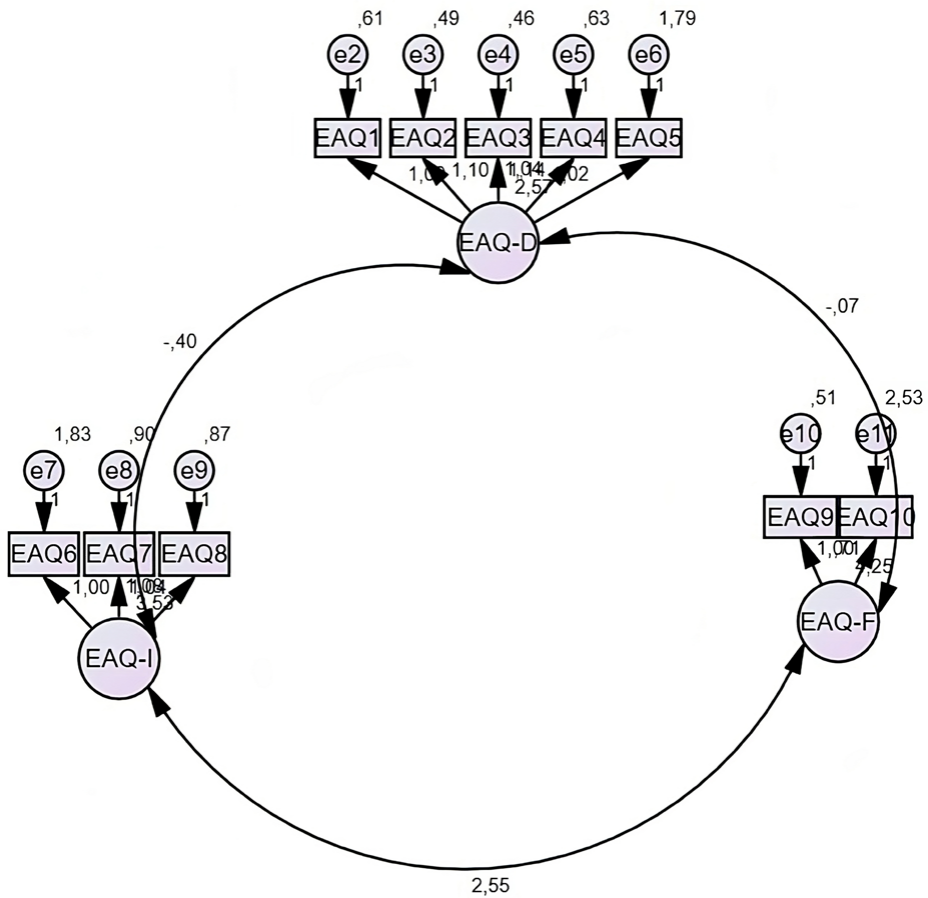
CFA chart of EAQ.

Table 4 and Figure 1 present the CFA statistics of the subdimensions of the EAQ. According to the CFA results, all items in the dimensions of EAQ‐D, EAQ‐I, and EAQ‐F had statistically significant and positive factor loadings (*p* < 0.001). In the CFA model presented in Figure 1, the relationship of each dimension with the items is visualized.

**TABLE 4:** CFA statistics of the subdimensions of the EAQ.

Subdimension	Question	*B*	SE (*B*)	*z*	*p*
Disgust (EAQ‐D)	EAQ1	1.000			
	EAQ2	1.099	0.028	38.674	**< 0.001**
	EAQ3	1.043	0.027	38.323	**< 0.001**
	EAQ4	1.140	0.031	37.335	**< 0.001**
	EAQ5	1.024	0.040	25.903	**< 0.001**
Interest (EAQ‐I)	EAQ6	1.000			
	EAQ7	1.081	0.040	27.015	**< 0.001**
	EAQ8	1.042	0.039	26.921	**< 0.001**
Feeding Animals (EAQ‐F)	EAQ9	1.000			
	EAQ10	0.709	0.051	14.027	**< 0.001**

Abbreviations: *B*, unstandardized regression coefficients; *p*, significance value.SE (*B*), standard error of *B*; *z*, test statistic. Bold values indicate statistically significant results at *p* < 0.05.

**FIGURE 2:**
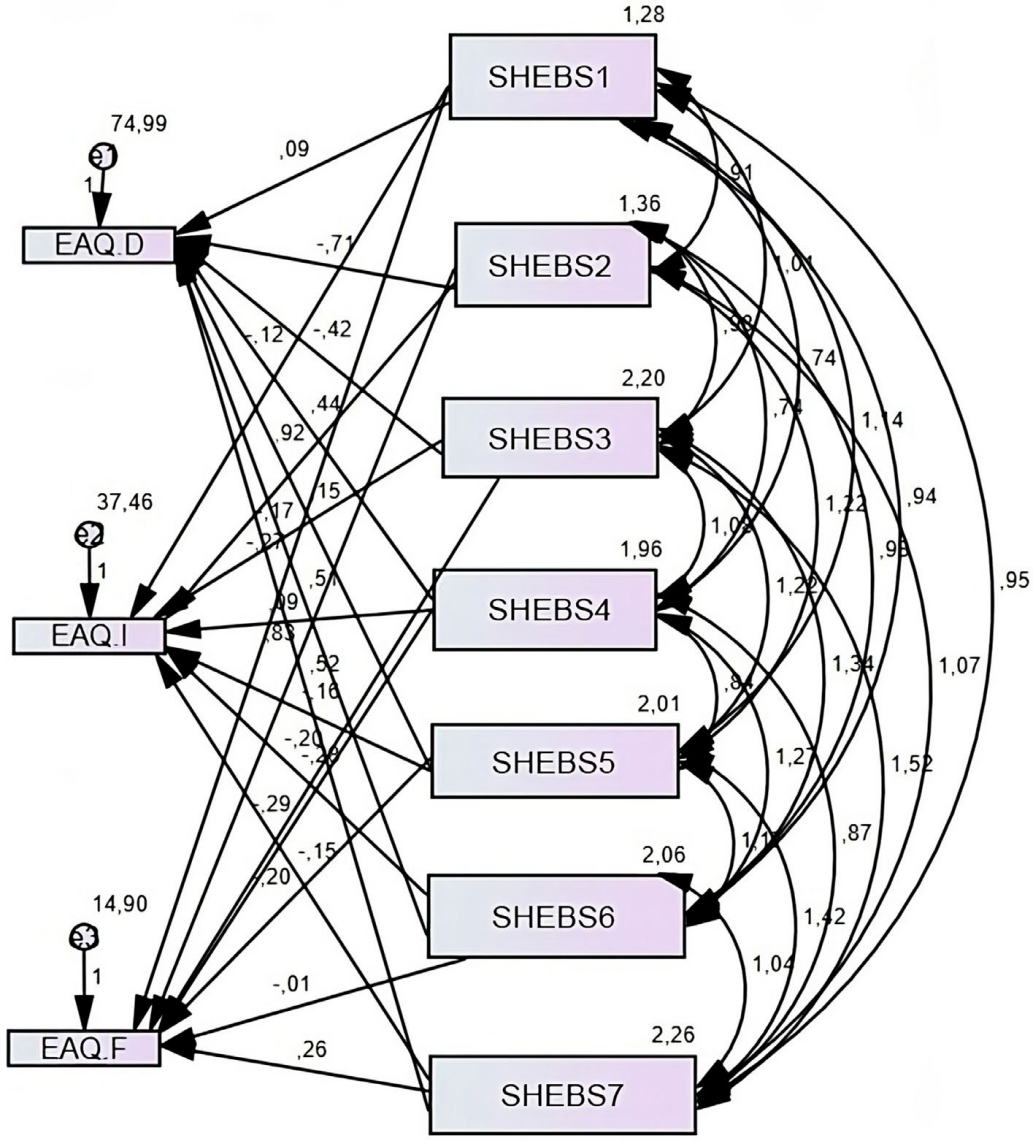
Structural equation model illustrating the relationships between SHEBS subdimensions (predictors) and EAQ subdimensions (outcomes). Path coefficients represent the strength of relationships. Statistically significant paths (*p* < 0.05) include ‘seasonal food and avoiding food waste’ positively influencing both EAQ‐Interest (EAQ‐I) and EAQ‐Feeding Animals (EAQ‐F), and ‘meat reduction’ negatively influencing EAQ‐Feeding Animals (EAQ‐F). All other paths shown are nonsignificant (*p* > 0.05). EAQ‐D, EAQ disgust; EAQ‐F, EAQ feeding animals; EAQ‐I, EAQ interest; SHEBS1, Quality labels (regional and organic); SHEBS2, Seasonal food and avoiding food waste; SHEBS3, Animal welfare; SHEBS4, Meat reduction; SHEBS5, Healthy and balanced diet; SHEBS6, Local food; SHEBS7, Low fat.

Table 5 and Figure 2 show the estimation results of the model examining the effect of SHEBS dimensions on EAQ. Structural equation modeling analyses showed that the SHEBS dimensions did not have a statistically significant effect on EAQ‐D (*p* > 0.05). Similarly, quality labels, animal welfare, meat reduction, healthy and balanced diets, local food, and low‐fat subdimensions did not have a statistically significant effect on EAQ‐I (*p* > 0.05). However, seasonal food and avoiding food waste dimensions had a positive and significant effect on the EAQ‐I dimension (*p* < 0.05). Similarly, quality labels, animal welfare, healthy and balanced diets, local food, and low fat dimensions have no significant effect on the EAQ‐F dimension (*p* > 0.05). However, seasonal foods and avoiding food waste have a positive effect on the EAQ‐F dimension, while the dimension of meat reduction has a negative effect on the EAQ‐F (*p* < 0.05). Table 5 also presents the fit indices of the model examining the effect of SHEBS on EAQ. According to the fit indices, *χ* ^2^/df = 3.645 is below 5, GFI, CFI, TLI, IFI, and AGFI values are above 0.90, and the RMSEA value is below 0.08.

**TABLE 5:** Estimates and fit indices of the structural equation model examining the effect of SHEBS subdimensions on EAQ subdimensions.

Dependent variable	Path	Independent variable	*B*	SE (*B*)	*z*	*p*
Disgust (EAQ‐D)	←	Quality labels (regional and organic)	0.011	0.060	0.177	0.859
	←	Seasonal food and avoiding food waste	−0.101	0.070	−1.448	0.148
	←	Animal welfare	−0.105	0.092	−1.140	0.254
	←	Meat reduction	0.148	0.108	1.364	0.173
	←	Healthy and balanced diet	0.037	0.105	0.352	0.725
	←	Local food	0.169	0.122	1.382	0.167
	←	Low fat	0.173	0.118	1.464	0.143
Interest (EAQ‐I)	←	Quality labels (regional and organic)	−0.015	0.043	−0.342	0.732
	←	Seasonal food and avoiding food waste	0.132	0.049	2.684	**0.007**
	←	Animal welfare	−0.042	0.065	−0.645	0.519
	←	Meat reduction	0.031	0.076	0.411	0.681
	←	Healthy and balanced diet	−0.039	0.074	−0.524	0.601
	←	Local food	−0.092	0.086	−1.061	0.289
	←	Low fat	−0.050	0.083	−0.597	0.551
Feeding Animals (EAQ‐F)	←	Quality labels (regional and organic)	−0.033	0.027	−1.243	0.214
	←	Seasonal food and avoiding food waste	0.118	0.031	3.799	**< 0.001**
	←	Animal welfare	−0.050	0.041	−1.232	0.218
	←	Meat reduction	−0.096	0.048	−1.981	**0.048**
	←	Healthy and balanced diet	−0.050	0.047	−1.069	0.285
	←	Local food	−0.002	0.055	−0.035	0.972
	←	Low fat	0.086	0.053	1.636	0.102

*χ* ^2^	df	GFI	AGFI	CFI	TLI	IFI	RMSEA
10.935	3	0.901	0.911	0.920	0.900	0.908	0.655

Abbreviations: *χ* ^2^, chi‐square; AGFI, adjusted goodness of fit index; *B*, unstandardized regression coefficients; CFI, comparative fit index; df, degrees of freedom; E (*B*), standard error of *B*; GFI, goodness of fit index; IFI, incremental fit index; *p*, significance value; RMSEA, root mean square error of approximation; TLI, Tucker–Lewis index; *z*, test statistic. Bold values indicate statistically significant results at *p* < 0.05.

**FIGURE 3:**
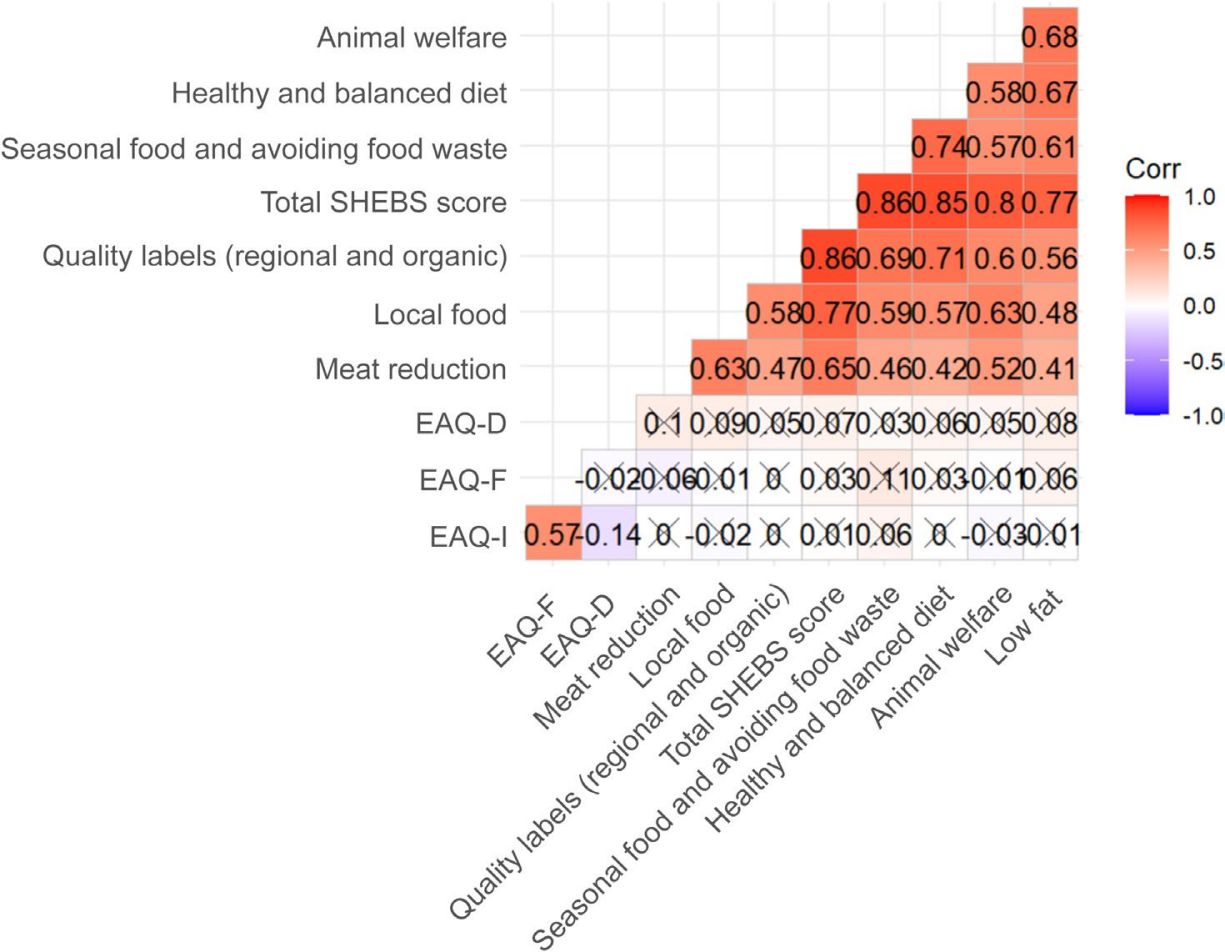
Relationships between the score points of SHEBS and EAQ. EAQ‐D, EAQ disgust; EAQ‐I, EAQ interest; EAQ‐F, EAQ feeding animals.

Figure 3 shows the correlations between SHEBS and EAQ dimensions. Positive and moderate to highly significant correlations were found between all subdimensions of SHEBS (*r* > 0.400, *p* < 0.05). The correlations between the EAQ subscales and SHEBS subscales were generally low, and most of them were not statistically significant (*p* > 0.05). A low positive correlation was observed between the dimension of seasonal foods and avoidance of food waste and the dimension of EAQ‐F (*r* = 0.107, *p* < 0.05). In addition, low positive correlations were found between the “local food” dimension and the EAQ‐D dimension (*r* = 0.092, *p* < 0.05) and between the “meat reduction” dimension and the EAQ‐D (*r* = 0.096, *p* < 0.05). Among the EAQ subdimensions, there is a moderate positive correlation between EAQ‐I and EAQ‐F (*r* = 0.570, *p* < 0.05). A negative correlation was found between EAQ‐D and EAQ‐I dimensions (*r* = −0.144, *p* < 0.05).

## 4 Discussion

This study aims to analyze the interaction between attitudes toward entomophagy and sustainable and healthy nutritional behaviors of Turkish Generation Z university students using structural equation modeling. The proposed model reveals how positive or negative approaches toward entomophagy are associated with sustainable and healthy nutritional practices and which factors significantly determine these attitudes. The model examines which sustainable dietary habits may shape young people's interest or resistance to insect consumption by revealing the connections between positive or negative attitudes toward entomophagy and certain dimensions of sustainable dietary behaviors. The research findings showed that students generally had negative attitudes toward insect consumption, but some sustainable nutritional habits may increase interest in entomophagy. In addition, validity and reliability analyses of the Turkish form of the EAQ were conducted in this study; the results revealed that the scale is a suitable, reliable, and valid tool for measuring entomophagy attitudes. This resistance may stem from traditional dietary norms, as suggested by Verneau et al. (2021). These findings are discussed in detail by comparing them with the results of other studies in the literature.

The demographic characteristics and environmental factors experienced by the 641 students participating in the study may be important factors affecting their attitudes toward entomophagy and sustainable eating habits. A notable proportion of the sample consisted of female students, which aligns with previous findings (Tuccillo et al. 2020; Lorini et al. 2021) suggesting that women often display more reservations about entomophagy due to health and environmental considerations. Most participants were in their early twenties, an age range often influenced by family habits and communal living conditions that can limit dietary exploration. Because many participants lived in dormitories or guesthouses, they often relied on ready‐made or traditional meals, making them less likely to explore alternatives such as entomophagy. Alghamdi et al. (2018) and İnce Palamutoğlu et al. (2024) stated that most of the students staying in dormitories have limited eating habits. In collective living spaces such as dormitories, individuals' dietary preferences are often based on group norms and ready‐to‐eat options, which may be a factor that creates resistance to different dietary alternatives, especially unusual options such as insect consumption.

Female participants showed significantly higher disgust scores compared to males (+0.81, *p* < 0.01). Future campaigns aimed at normalizing entomophagy may therefore benefit from emphasizing strict food safety measures, transparent production practices, and cosmetic‐free formulations, which have been shown in previous research to reduce aversive reactions, particularly in female consumers.

Among students with health conditions that heighten concerns about food safety, reservations toward entomophagy may be amplified. This may be considered a reason for the negative attitude toward entomophagy. Chronic diseases may cause individuals to be more sensitive about food safety and health effects. Therefore, the acceptance of insects as food may increase health concerns. Students with greater health knowledge, particularly those in health‐related disciplines, may develop more pronounced reservations about entomophagy. A high level of health knowledge may lead students to perceive insect consumption as a risk factor and trigger feelings of disgust. Health education may increase individuals' awareness of food safety and potential health risks of food. This may lead them to develop a more cautious and negative attitude toward the consumption of insects. These findings are confirmed in other studies in the literature; for example, resistance to entomophagy is directly related to individuals' health and safety concerns (Belluco et al. 2013; Lange and Nakamura 2021; Conway et al. 2024). In conclusion, the findings based on the demographic and living conditions of the students indicate that environmental, health‐related, and cultural factors may be effective in shaping their attitudes toward entomophagy. These findings suggest that alternative dietary options, such as entomophagy, should be promoted more appropriately to encourage sustainable and healthy eating behaviors, considering health and safety perceptions for wider acceptance in society.

The EAQ data indicate a stronger tendency toward disgust relative to interest, suggesting an overall reluctance to adopt insect consumption. The findings obtained overlap with previous studies (Caparros Megido et al. 2014; Cicatiello et al. 2016; Peksever et al. 2024), which indicate that resistance and disgust toward entomophagy are strong in Western societies. However, it has been stated that familiarity and exposure can change such negative perceptions. Caparros Megido et al. (2014) found that Belgian consumers were more willing to try insect‐based products when presented in familiar forms or paired with known flavors. Verneau et al. (2021) also found that the level of disgust decreased and interest increased in individuals with previous experience in insect consumption, indicating that exposure plays an important role in shaping attitudes. This suggests that familiarity and positive experiences with insect consumption may play an important role in reducing disgust and increasing acceptance.

The findings of Caparros Megido et al. (2014) suggest that even a single exposure to insect‐based foods can positively influence attitudes by reducing neophobia and increasing future willingness to consume insects. Given the high baseline disgust scores observed in our sample, future studies could test whether repeated sensory exposures produce cumulative desensitization effects over time–particularly if integrated into structured, culturally sensitive formats. This would inform the optimal design and sequencing of sensory‐based interventions in university populations.

International comparisons allow us to place Turkish consumer attitudes toward entomophagy in a broader context. In our study, the mean disgust score (EAQ‐D = 5.90 ± 1.75) was significantly higher than that reported in both Chilean (EAQ‐D = 4.18 ± 1.76) and Italian samples (EAQ‐D = 4.96 ± 1.75) using the same EAQ tool (La Barbera, Verneau, et al. 2021; La Barbera, Amato, et al. 2021; Sogari et al. 2023). Turkish participants also showed less curiosity about tasting insects, with an interest score (EAQ‐I = 2.61 ± 2.06) well below the scores in Chile (3.46 ± 1.87) and Italy (3.49 ± 1.94). The Feeding Animals subscale (EAQ‐F), which reflects the acceptance of insects as animal feed, also followed this pattern: 3.50 ± 1.97 in our sample versus 4.69 ± 1.82 in Chile and 4.16 ± 1.49 in Italy. These attitudinal differences may be influenced by a combination of factors such as religious dietary laws (e.g., halal concerns), lack of market exposure to edible insect products, and cultural unfamiliarity. The relatively high levels of disgust and low levels of interest scores in our Turkish student sample reinforce the idea that socio‐cultural framing plays a key role in shaping entomophagy acceptance.

The EAQ‐F subscale indicated a moderate openness to using insects in animal feed, implying that acceptance may be somewhat higher when insects are consumed indirectly. This finding is supported by the literature on the acceptance of adding insects to animal feed. NaranjoGuevara et al. (2021) reported that when students were provided with information on the benefits of insects to animal health and the environment, these benefits increased their willingness to use insects in animal feed. These findings suggest that positive attitudes toward the use of insects as animal feed can be strengthened by increasing knowledge and awareness on this issue.

The basic assumptions of the EFA process of the EAQ and SHEBS scales were evaluated with KMO and Bartlett's test of sphericity (Table 3). Bartlett's test of sphericity results indicate that there is a significant correlation between the items for both scales, indicating that there is a sufficient level of relationship for factor analysis. The KMO values were 0.859 and 0.94, respectively, confirming that both scales were suitable for factor analysis in terms of sample size. The reliability indicators for EAQ‐D and EAQ‐I were notably high, suggesting strong consistency in measuring disgust and interest. Meanwhile, EAQ‐F, though acceptable, may require further refinement in future studies. The fit indices for the CFA findings of the EAQ and SHEBS are also presented in Table 3. A fit index value of *χ* ^2^/df below 5 for EAQ indicates that the model has an acceptable fit. GFI, AGFI, CFI, TLI, and IFI values above 0.90 indicate that the model provides a good fit. The RMSEA value is 0.070, which is within the acceptable fit limits as a value below 0.08 (Polat et al. 2023). These findings suggest that the Turkish version of the EAQ is a reliable and valid instrument for measuring attitudes toward entomophagy. Similar fit criteria were met for the fit index values for SHEBS. These results indicate that SHEBS has an acceptable fit. In general, based on these fit indices, it can be concluded that the construct validity of both scales has been achieved and can be accepted as a valid measurement tool. These findings reveal that both scales are reliable and valid in assessing attitudes toward the related concepts (Polat et al. 2023).

According to the CFA results, all items in the dimensions of EAQ‐D, EAQ‐I, and EAQ‐F had statistically significant and positive factor loadings (*p* < 0.001), supporting the construct validity of the model (Table 4 and Figure 1). These findings confirm that each dimension of the questionnaire shows a strong agreement with the relevant items and that the scale provides a valid construct for measuring attitudes toward entomophagy. In particular, the high path coefficients of the items in the EAQ‐D and EAQ‐I dimensions emphasize the decisive influence of these dimensions on entomophagy attitudes, while the relatively low factor loading (*B* = 0.709) of the EAQ10 item in the EAQ‐F dimension may indicate that the contribution of this item should be reviewed in future studies. The structure presented in Figure 1 supports the internal consistency and the accuracy of the factor structure of the scale and demonstrates the validity of the Turkish version of the questionnaire.

According to the fit indices of the model examining the effect of SHEBS on EAQ (Table 5), *χ* ^2^/df is below 5, GFI, CFI, TLI, IFI, and AGFI values are above 0.90, and the RMSEA value is below 0.08. These findings indicate that the structural equation model examining the effect of SHEBS on EAQ has an acceptable fit. In general, SHEBS showed a low level of correlation with attitudes toward entomophagy; it was observed that most of the subdimensions did not have a statistically significant effect (Table 5). Notably, those who prioritize seasonal foods and waste reduction appear more open to entomophagy, potentially due to heightened sustainability awareness. This finding suggests that individuals with sustainable nutrition awareness may be more open to insect consumption. Woolf et al. (2019) reported that participants who were aware of the environmental benefits of entomophagy were more willing to consume insects. Similarly, Mancini et al. (2019) stated that educational seminars and information provision programs on entomophagy can increase consumer acceptance, and emphasizing the environmental benefits of insect consumption can strengthen these attitudes. In line with these findings, it is thought that raising awareness about the ecological advantages of insect consumption can bridge the gap between sustainable practices and entomophagy acceptance. On the other hand, it was observed that other dimensions of SHEBS (quality labels, animal welfare, meat reduction, etc.) had no statistically significant effect on EAQ‐F and EAQ‐D toward entomophagy (*p* > 0.05) (Table 5). This lack of association is consistent with previous findings by Lammers et al. (2019) and Laureati et al. (2016), who reported that sustainability awareness was not a direct predictor of willingness to consume insects. These findings also suggest that while general sustainability awareness may not predict acceptance of entomophagy, specific behaviors—such as prioritizing seasonal foods and avoiding food waste—can significantly increase openness to consuming insects or using them as animal feed. Interestingly, students with strong meat reduction orientations showed a reduced willingness to support insect‐based animal feed, revealing a paradox where ideologically motivated dietary changes do not always align with novel sustainable food solutions. These findings highlight the importance of designing communication strategies that focus not only on general sustainability values but also on specific, actionable behaviors that have been shown to promote the acceptance of entomophagy.

However, two findings in our study deviated from these expectations and may warrant further theoretical consideration. First, students who reported a strong commitment to seasonal food consumption and avoiding food waste showed significantly greater interest in entomophagy. This suggests that sustainability‐oriented values may translate into increased curiosity about novel protein sources such as edible insects—an insight that supports targeted messaging strategies. Second, and somewhat unexpectedly, students with stronger meat reduction orientations showed a lower willingness to use insects as animal feed. This paradox may reflect a broader ideological rejection of all animal‐based inputs, including those perceived as novel or industrial. These counterintuitive findings may inform future hypotheses on how different sustainability profiles shape attitudes toward entomophagy. Furthermore, given that the Feeding Animals subscale (EAQ‐F) showed relatively lower internal consistency (Cronbach's alpha = 0.780) compared to other subscales, future research could explore potential item modifications or additions to enhance its psychometric robustness.

These findings can also be explained by the participant's lack of knowledge about the contribution of insects to sustainability. Similarly, Bakkaloğlu (2022) stated that the participants could not establish a strong link between sustainability and insect consumption because they did not have sufficient knowledge about entomophagy. In conclusion, these findings reveal the importance of increasing the level of knowledge to promote insects as a sustainable food source. In particular, educational programs on the nutritional and environmental benefits of food can improve individuals' attitudes toward entomophagy and contribute to the adoption of sustainable eating habits. Such education can help individuals overcome their resistance to insect consumption and accept healthier, sustainable eating habits.

Based on our findings, educational interventions to promote the acceptance of entomophagy in Türkiye should prioritize culturally sensitive and science‐based communication strategies. Given the significant influence of traditional dietary norms and religious considerations, campaigns should initially focus on presenting insect‐based foods as environmentally sustainable and scientifically safe, rather than attempting to directly alter culinary identities. Structured seminars led by nutritionists, food scientists, and religious scholars can be organized within universities to present evidence on the environmental benefits, nutritional value, and potential religious acceptability (halal status) of edible insects. These seminars should be complemented by carefully curated outreach materials—such as brochures and visual posters—that highlight lifecycle assessments comparing insects with conventional livestock, with an emphasis on food security, waste reduction, and climate change mitigation. In addition, integrating theoretical modules on novel protein sources into elective university courses on public health nutrition and sustainability would allow students to critically engage with the topic in a nonconfrontational academic setting. Pilot sensory sessions should only be considered after basic awareness has been established, thereby reducing the risk of early negative sensory bias. All campaigns should frame insect consumption as an environmentally responsible supplement rather than as a radical dietary change, ensuring that communication remains respectful of prevailing cultural sensitivities (Mancini et al. 2019). In practical terms, collaboration with major retail chains and supermarkets in Türkiye to introduce limited‐time promotional activities or demonstration booths featuring edible insect products could also significantly increase exposure and gradually normalize these foods within the local market.

Correlation analysis between the SHEBS and EAQ dimensions showed that sustainable eating habits generally had a low association with attitudes toward entomophagy (Figure 3). This suggests that entomophagy in the context of sustainability is still limited in terms of social acceptance and prevalence and that the link between entomophagy and sustainable nutrition is not fully recognized (Li 2023; Olivadese and Dindo 2023). A slight positive association between sustainability practices and willingness to use insects as feed suggests that individuals mindful of environmental issues might view entomophagy more favorably in indirect consumption contexts. This finding implies that sustainable feeding behaviors are more open to potential applications of entomophagy (Varelas 2019; van Huis & van Huis and Gasco 2023). Additionally, the fact that the “local food” dimension showed low positive correlations with EAQ‐D and the “meat reduction” dimension showed low positive correlations with EAQ‐D indicates that individuals' preferences for reducing local and meat consumption are parallel to their disgust toward entomophagy. This suggests that interest in local and alternative protein sources should be evaluated together with prejudices against entomophagy and that breaking these prejudices may increase the acceptance of entomophagy (Onwezen et al. 2021).

The moderately positive correlation between the EAQ‐I and EAQ‐F dimensions (Figure 3) suggests that individuals who are interested in entomophagy are also prone to using insects as animal food. This finding is in line with studies in the literature indicating that providing information on the health and environmental benefits of insects may increase acceptance (Caparros Megido et al. 2014; Mancini et al. 2019). In particular, it suggests that interest in entomophagy may encourage the use of such alternative protein sources in both human and animal diets. The finding of a negative correlation between EAQ‐D and EAQ‐I dimensions indicates that the feeling of disgust toward entomophagy is inversely proportional to the level of interest. The decrease in negative attitudes toward entomophagy may have a direct effect on the increase in interest. Therefore, reducing negative perceptions toward entomophagy in society and raising awareness may be a critical strategy for increasing interest in this orientation.

### 4.1 Strengths and Limitations

This study has some important limitations. First of all, since the study was conducted in a single center at (blinded for review) University and with participants in the age group of 18–23, it is difficult to directly generalize the findings obtained to different geographical regions or wider age ranges. Secondly, since the study has a cross‐sectional design, although it can reveal the relationship between the variables, it does not allow definite inferences to be made about the causality (cause‐effect) relationship. Thirdly, collecting data through self‐reported questionnaires may introduce the risk of social desirability bias, which may affect the results by increasing the tendency of respondents to give more favorable or socially acceptable responses than they actually do. Finally, attitudes toward a relatively little‐known topic such as insect consumption vary depending on many factors such as cultural norms and individuals' past experiences and tastes; therefore, further studies in different universities, age groups, or cultural contexts may provide a more comprehensive view on the validation of the findings and acceptance of entomophagy.

## 5 Conclusion and Recommendations

This study revealed that the attitudes of Turkish Generation Z university students toward entomophagy have a limited relationship with some sustainable nutritional behaviors. The findings show that negative attitudes toward entomophagy are widespread and that awareness‐raising training can play an important role in changing this attitude. It is thought that educational programs and information campaigns that emphasize the sustainability, environmental, and health issues of insects can increase the acceptance of entomophagy. In this context, increasing the level of knowledge about the potential benefits of entomophagy can be an important factor that can change students' nutritional habits and attitudes. Education and awareness‐raising studies can contribute to the acceptance of alternative nutritional options such as insect consumption by wider audiences and the spread of sustainable nutritional habits.

It is important and necessary to use innovative and inclusive educational methods in studies on this subject. In this context, presenting entomophagy as an option that contributes to healthy and sustainable nutritional habits can be effective in changing social attitudes. It is thought that education and awareness studies will encourage sustainable nutritional practices while increasing interest in entomophagy with innovative and accessible methods.

Given the cultural and regulatory landscape in Türkiye, several targeted actions are recommended to facilitate the safe and acceptable introduction of insect‐based products: (i) establishing an accelerated approval pathway for novel foods, especially insect‐derived ingredients; (ii) providing financial incentives or subsidies for the use of insect meal in poultry and aquaculture feeds to achieve economies of scale; and (iii) supporting the development of public‐private incubators to promote scalable, safe, and culturally appropriate insect farming initiatives. In addition, regulatory authorities such as the Ministry of Agriculture and Forestry could establish clear and detailed guidelines on acceptable insect species, processing standards, labeling requirements, and market authorization procedures, thus providing a structured pathway for the development of insect‐based products.

Future experimental studies should use factorial designs to isolate the mechanisms driving attitude change. For example, a 2 × 2 factorial design could cross message framing (environmental vs. sensory benefits) with sensory exposure (visual imagery only vs. actual tasting). This approach would allow researchers to disentangle the relative influence of informational versus experiential factors and provide causal evidence on the most effective strategies for promoting entomophagy acceptance.

## Author Contributions

**Emre Duman:** conceptualization (equal), data curation (equal), investigation (equal), resources (equal), validation (equal), visualization (equal), writing – original draft (equal), writing – review and editing (equal). **Alev Keser:** conceptualization (equal), data curation (equal), investigation (equal), methodology (lead), project administration (lead), resources (equal), supervision (lead), validation (equal), visualization (equal), writing – original draft (equal), writing – review and editing (equal).

## Ethics Statement

Ethical approval for the involvement of human subjects in this study was granted by Ankara University Research Ethics Committee, Reference number 09/71, dtd 05/29/2023.

## Conflicts of Interest

The authors declare no conflicts of interest.

## Data Availability

The data that support the findings of this study are available from the corresponding author upon reasonable request.
