# Correction to “Entomophagy Attitudes Among Turkish Generation Z University Students: A Scale Validation and Path Analysis Model for Sustainable and Healthy Dietary Choices”

**DOI:** 10.1002/fsn3.70526

**Published:** 2025-07-02

Duman, E. and Keser, A. (2025), Entomophagy Attitudes Among Turkish Generation Z University Students: A Scale Validation and Path Analysis Model for Sustainable and Healthy Dietary Choices. Food Sci Nutr, 13: e70397. https://doi.org/10.1002/fsn3.70397

In the published article, six instances of the placeholder text “(blinded for review)” remained in Sections 2.2, 2.3, and 4.1. The sentences should read as follows:

Section 2.2 (Participants)

“…studying at **Ankara** University, Türkiye.”

“The ethical approval required for the research was obtained from the **Ankara** University Research Ethics Committee with the permission dated **05/29/2023, Reference number 09/71**.”

“The exclusion criteria for this study were as follows: (1) students studying outside **Ankara** University; (2) individuals under 18 or over 23 years of age; (3) vegan or vegetarian participants; and (4) students enrolled in nutrition and dietetics, food engineering, or gastronomy programs.”

Section 2.3 (Data Collection Tools)

“As a result of the decision made by **Ankara** University to conduct the courses by distance education (online) method due to the ongoing COVID‐19 pandemic in the 2022–2023 spring semester, the study questionnaire was applied using the online Google Forms platform.”

Section 4.1 (Strengths and Limitations)

“This study has some important limitations. First of all, since the study was conducted in a single center at **Ankara** University and with participants in the age group of 18–23, it is difficult to directly generalize the findings obtained to different geographical regions or wider age ranges.”

Additionally, the following funding acknowledgment was inadvertently omitted and should be added:

**Funding:** Open access funding provided by the Scientific and Technological Research Council of Türkiye (TÜBİTAK).

We apologize for these errors.

